# Stability and change: Stress responses and the shaping of behavioral phenotypes over the life span

**DOI:** 10.1186/1742-9994-12-S1-S18

**Published:** 2015-08-24

**Authors:** Michael B Hennessy, Sylvia Kaiser, Tobias Tiedtke, Norbert Sachser

**Affiliations:** 1 Department of Psychology, Wright State University, Dayton OH 45435, USA; 2 Department of Behavioural Biology, University of Muenster, Badestrasse 13, 48149, Muenster, Germany

**Keywords:** early experience, stress, maternal separation, social buffering, behavioral adaptation, social behavior, hypothalamic-pituitary-adrenal, cortisol, immune, cytokines, guinea pig

## Abstract

In mammals, maternal signals conveyed via influences on hypothalamic-pituitary-adrenal (HPA) activity may shape behavior of the young to be better adapted for prevailing environmental conditions. However, the mother's influence extends beyond classic stress response systems. In guinea pigs, several hours (h) of separation from the mother activates not only the HPA axis, but also the innate immune system, which effects immediate behavioral change, as well as modifies behavioral responsiveness in the future. Moreover, the presence of the mother potently suppresses the behavioral consequences of this innate immune activation. These findings raise the possibility that long-term adaptive behavioral change can be mediated by the mother's influence on immune-related activity of her pups. Furthermore, the impact of social partners on physiological stress responses and their behavioral outcomes are not limited to the infantile period. A particularly crucial period for social development in male guinea pigs is that surrounding the attainment of sexual maturation. At this time, social interactions with adults can dramatically affect circulating cortisol concentrations and social behavior in ways that appear to prepare the male to best cope in its likely future social environment. Despite such multiple social influences on the behavior of guinea pigs at different ages, inter-individual differences in the magnitude of the cortisol response remain surprisingly stable over most of the life span. Together, it appears that throughout the life span, physiological stress responses may be regulated by social stimuli. These influences are hypothesized to adjust behavior for predicted environmental conditions. In addition, stable individual differences might provide a means of facilitating adaptation to less predictable conditions.

## Introduction

One of the most well-established principles in the study of psychobiological development is that exposure to stressors during the earliest stages of life can dramatically affect behavior and physiological processes at later life stages [1-3]. For the newborn mammal, these early experiences are often inextricably linked to the infant's interactions with its mother. Separation from the mother or variations in the way she treats her young can alter later behavioral and physiological processes. On the other hand, active care-giving or the mother's mere presence can moderate the impact of stressful events [4,5]. In recent years, increasing recognition that the mother also serves as a potential source of information concerning the environmental circumstances in which the offspring are likely to develop has re-focused attention on the role the mother can play in shaping the young's biobehavioral development to better adapt to future environmental demands. Indeed, the mother's interactions with her offspring can profoundly influence stress physiology, and thereby the young's developmental trajectory [6,7]. The most commonly examined aspect of the infant's modified stress physiology is that of the hypothalamic-pituitary-adrenal (HPA) axis, particularly changes in glucocorticoid responses [1,8]. The mother's behavior can alter neural mechanisms regulating later glucocorticoid secretion; and altered corticoid responsiveness is an effective tool for lasting biobehavioral change [9,10]. Yet, glucocorticoid action is not the only mechanism for such change; the mother is not the only social partner that can exert such influence; and the neonatal period is not the only life stage at which such influences can be exerted.

In this paper, we will first address how the presence or absence of the mother potently and selectively impacts immunological activity in addition to HPA and other classic stress responses, and how the immunological changes mediate behavior in the short term. We will then discuss longer-lasting immunological changes in response to maternal separation, and how these changes may serve as a mechanism for shaping future adaptive behavioral phenotypes. Next, we will consider the effect of other social partners during later life stages, and how social influences on HPA activity during the adolescent period appears to promote behavior that is adaptive in specific environments. Finally, we will explore an issue not usually addressed in discussions of socially-induced modifications of physiological stress responses; that is, whether inter-individual consistency in these responses is maintained in the face of social modification at various life stages.

To examine these questions, we will focus primarily, though not exclusively, on studies of the guinea pig, a gregarious species well-suited for this purpose. We probably know more about the behavioral and endocrine profiles of the guinea pig throughout the lifespan than those of any other laboratory rodent. Data from the wild ancestor brought into the laboratory indicate that the guinea pig has gone through fairly typical changes associated with domestication (e.g., increased courtship, reduced HPA responses), but no obvious radical or qualitative alterations in behavior or physiology (Kaiser et al., this volume). This allows us to at least tentatively place our findings in an evolutionary perspective, even though much basic information on the behavior and ecology of the wild species remains unknown. We are, therefore, viewing the guinea pig as a model species, and while the findings we review have largely been identified only in the guinea pig to this point, we propose they indicate in principle how the shaping of behavioral phenotypes might occur more generally.

Research in the guinea pig, perhaps more than in any other mammal, has documented continuing reciprocal influences between social behavior and physiological stress responses throughout the life span. Infants exhibit evidence of a specific attachment to their mothers [11-13] and adult males form social bonds with particular breeding partners [14,15]. Moreover, the social organization of the guinea pig is complex and varies with environmental conditions, such that the breeding success of males relies on differing behavioral strategies under differing social conditions [3,14]. Although the present paper concerns the consequences of direct exposure to stressors, it should be also noted that guinea pigs are indirectly sensitive to stressors even before birth, when the relative stability of social conditions experienced by the mother produces long-term, sex-specific consequences that appear to better adapt the offspring for particular environmental contingencies (see [16] for review).

## Maternal influences on endocrine, immune, and behavioral stress responses

*Responses during maternal separation.* Guinea pig pups are born in an advanced developmental state. They can locomote from shortly after parturition, nibble on solid food and drink from a water spout within 24 h, and readily be raised without the mother from birth [17]. Maternal behavior is passive—mothers do not retrieve pups and engage in almost no active interaction with the pup (e.g., licking) beyond the first week of life [18]. Pups initiate virtually all nursing bouts [19], with mothers merely allowing access to the nipple. Nursing continues until about 3-4 weeks of age. Throughout the preweaning period it is the responsibility of the pup to follow and maintain proximity with the mother.

Despite its physical maturity, if a pup of about 2 weeks of age is simply placed alone into a brightly lit novel cage, there is immediate activation of the major physiological stress systems. Plasma epinephrine and norepinephrine levels are elevated, and there is increased central turnover of norepinephrine and dopamine [20-22]. This isolation procedure also produces clear activation of the HPA axis [15,21,23], including increased corticotropin-releasing factor gene expression in the paraventricular nucleus, and elevations in circulating levels of ACTH and cortisol (the primary glucocorticoid of guinea pig adrenal). None of these changes occur if the mother is placed into the cage with the pup. Thus, the presence or absence of the pup's maternal attachment figure determines whether classic physiological stress responses are exhibited in this situation.

While these effects have all been known for some time, recent evidence suggests separation in the same paradigm also activates the innate immune system. When one considers activation of immune responses, it typically is in the context of pathogen exposure. If monocytes or macrophages circulating in the periphery detect viruses, bacteria, or other pathogens, they secrete an array of peptide messengers referred to generically as cytokines. Proinflammatory cytokines stimulate a nonspecific systemic inflammatory state, sometimes designated simply as “sickness”, that combats a broad range of pathogens. Sickness is characterized by, among other reactions, an increase in core body temperature set point, or fever. Fever promotes further immune activation and inhibits pathogen replication [24] and so is adaptive in this situation. But sickness also has behavioral consequences. Peripheral cytokines enter the brain or initiate neural or vascular signals [25] that stimulate brain cells, primarily microglia, to release their own array of cytokines. These peptides then act on limbic and other regions, largely in a neuromodulatory fashion [26], to produce “sickness behaviors”. Some of these behaviors, such as seeking warmth, piloerection, shivering, and assuming a hunched posture generate or conserve body heat, and thus support fever. Behaviors such as hypersomnia, reduced environmental exploration, and loss of social motivation conserve energy needed for increased thermogenesis [24] and may prevent spread of illness to conspecifics. Of particular relevance here, a number of stressful events, such as footshock, restraint, and defeat can also stimulate sickness responses, including cytokine release, fever, and sickness behaviors [27-30].

Guinea pig pups, like the young of some primate species, display a two-stage, active/passive behavioral response during maternal separation [31,32]. After about an hour of active behavior, primarily vocalizing, pups quiet and display a characteristic crouched stance with prolonged eye-closure and extensive piloerection—behaviors that are indicative of sickness. This second stage can be greatly enhanced by direct activation of a proinflammatory cascade [33]. Furthermore, separation has been found to increase physiological signs of an inflammatory reaction, specifically increased proinflammatory cytokine expression and fever [34,35]. Finally, several anti-inflammatory compounds reduce the level of the passive behaviors exhibited by separated infants [36-38]. Together, these findings provide strong evidence that the behaviors of the second stage are examples of stress-induced behavior mediated by proinflammatory activity.

*The adaptive role of immune activation during separation.* Unlike during pathogen exposure, however, it is difficult to argue that the crouching, prolonged eye-closure, and piloerection of separated pups serve to support fever. The fever response during separation is brief, peaking around 90 min and dissipating by about 3 h, while the passive behaviors are still evident after 24 h of separation [31,35,39]. Rather, it appears that cytokine signaling is involved in the initiation of the passive response, but some other factors must contribute to its maintenance. In this regard, it may be instructive to remember that cytokines are peptides that are active neurally, as well as immunologically. Further, cytokines now are known to be broadly involved in basic neural functions such as brain development, synaptogenesis, and long-term potentiation [40]. In other words, central proinflammatory cytokine activity appears to be another basic physiological stress response that is elicited by the guinea pig separation procedure, and one that contributes to the behavioral response exhibited by separated pups.

If the passive response does not function to support fever, does it have adaptive significance during separation? Many years ago, Kaufman and Rosenblum [41] proposed that the passive second stage of separation exhibited by pigtailed macaque infants could be accounted for with the ”conservation/withdrawal” hypothesis. It was argued that if an initial period of vocalizing and searching did not produce reunion with the mother, it would be more adaptive for the infant to quiet, conserve energy, and wait for the mother's return. A similar interpretation may be applied to the separated guinea pig. In the natural habitat, the domestic guinea pig's wild progenitor (*Cavia aperea*) is widely preyed upon by mammalian, reptilian, and avian predators [42]. The wild guinea pigs often feed in open meadows [42], and the young follow and forage with the mother [43]. If a young guinea pig were to find itself alone in a threatening situation from which it could not escape (as the brightly lit test environment may simulate), and vocalizing did not re-establish contact with the mother, it might be adaptive for the pup to remain quiet and inconspicuous until the mother returned so as to conserve energy and not attract predators.

Of course, this scenario is quite speculative, but if it were to be the case, it would also be necessary for the return of the mother to quickly suppress the passive response so that the young could follow her. This, indeed, is what has been found. Pups were first isolated for 2 h to allow the underlying immune response to build and passive behavior to emerge. They then either had the mother returned to the cage or the experimenter simply reached into the cage as a control. The return of the mother immediately and consistently suppressed passive behavior of the pups for the entire 60-min observation period [44]. The potency of the mother's inhibitory influence is such that her presence even suppressed passive behavior driven by activating cytokine release pharmacologically [44]. Further, this effect may be exclusive to the mother. When placed into a novel environment with either a littermate, adult male, or an unfamiliar adult female, passive behavior increased to the same extent as when the pup was alone [45,46]. In all, the findings suggest that the function of these behaviors in separated pups has more to do with mother-infant interactions than with processes such as fever. In short, a system evolved for combating pathogens may have been co-opted for producing adaptive behavioral responses in isolated young. It would appear that the stress-induced cytokine release of separated guinea pig pups is one of a number of factors that help orchestrate the behavioral response in this situation: Cytokines seem to initiate the passive response, but other mechanisms are both responsible for its maintenance and can quickly suppress passive behavior when it no longer appears to be serving an adaptive function.

## Early postnatal stress exposure and the shaping of behavioral phenotypes

A growing body of literature has documented potentially adaptive consequences of early life stressors [47-49]. Because the surroundings into which an infant is born are often the best predictors of its future environment, early experiences may shape the individual in ways that will help it best cope with later challenges of various kinds [50,51]. The predominant hypothesized mediator of these effects is glucocorticoid activity. It has been known since the middle of the last century that challenging conditions during the preweaning period can modify HPA responsiveness in adulthood [52]. Early studies first demonstrated that removing infant rats or mice from the home cage for short or more prolonged periods, or administering additional stressors during separation, had widespread later effects on both HPA responsiveness and behavior. Recent studies have extended our understanding of how altered HPA hormone levels might modify the behavioral phenotype to be adaptive in later life [53-56].

While scores of studies have investigated effects of stressors on the infant’s later HPA activity, a smaller set of studies with rats and mice has addressed the possibility that activation of the innate immune system at the same age might produce neural, endocrine, and behavioral effects similar to those produced by the early stressors. The immune activation is induced by injection of lipopolysacchride (LPS)—which is derived from the cell-wall of gram-negative bacteria—or by exposure to a live pathogen [57,58]. Indeed, experiments using LPS, which allows estimation of effects of early immune activation free from confounding influences of a replicating pathogen, have found behavioral and HPA effects in rats that parallel those of infantile stressors. Preweaning injection with LPS as well as prolonged maternal separation increased HPA responsiveness in later life. The mechanism in each case appears to involve a reduction in hippocampal and other central glucocorticoid receptors, which reduces HPA negative feedback, ultimately resulting in increased hypothalamic secretagogue levels (corticotropin-releasing factor and arginine vasopressin) to provide greater excitatory input to the pituitary under stressful conditions in adulthood [59]. Some similarities in adult behavioral effects of early LPS exposure and prolonged maternal separation have also been reported, including increased anxiety-like behavior in the elevated plus maze [60,61] and impaired active avoidance learning [62,63]. These findings are important not only because they demonstrate that stimuli that activate the immune system can have long-lasting effects comparable to those of commonly studied early stressors, such as maternal separation, but also because they raise the possibility that early *stressors* might exert some of their long-term adaptive consequences through the release of cytokines.

**Figure 1:**
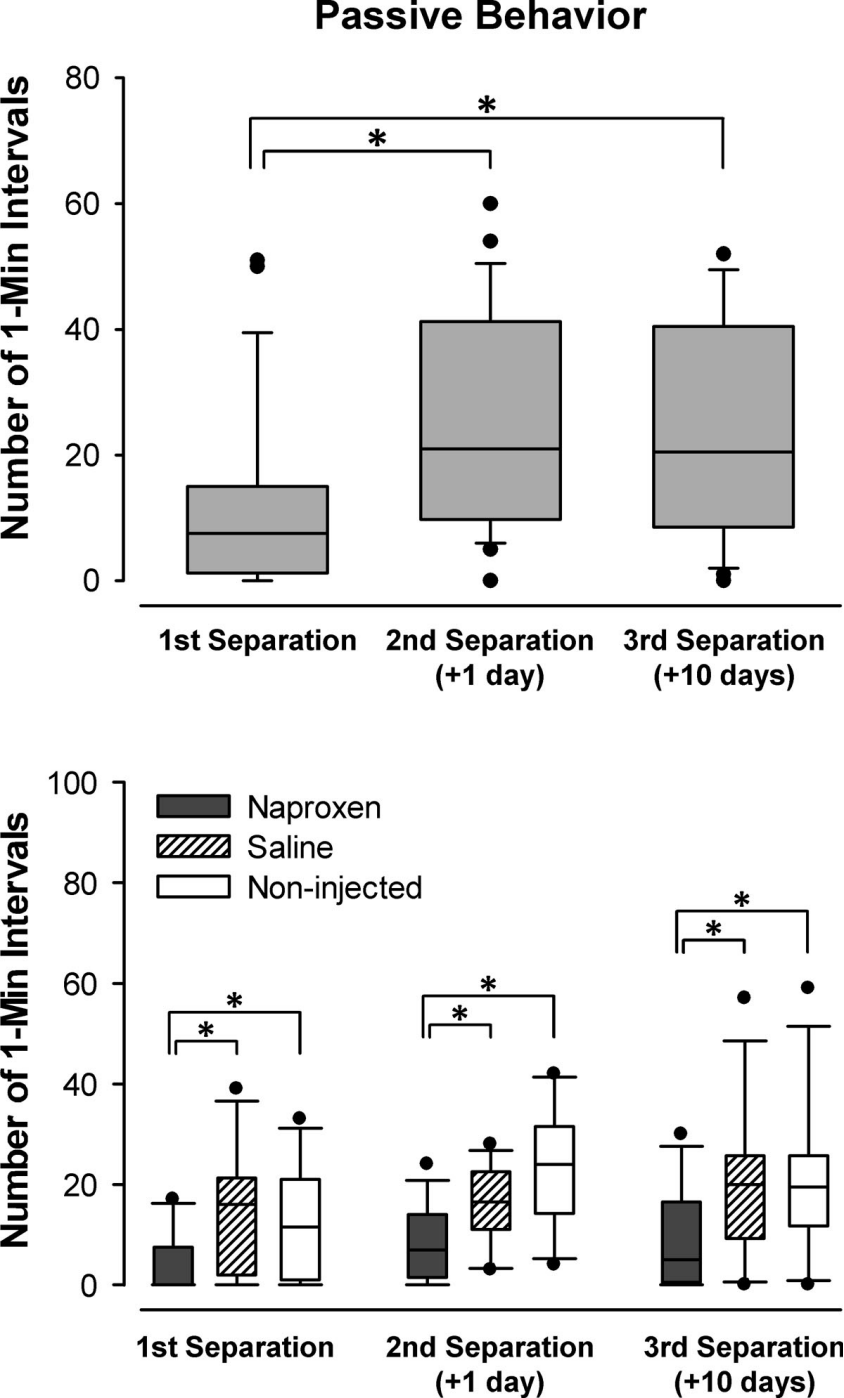
Median (and box plots) of the number of 1-min intervals in which young guinea pigs exhibited the passive behaviors of crouching, eye-closure, and extensive piloerection during 3-h separations in a novel enclosure during two consecutive days just prior to weaning and then again 10 days after the first day. The top panel illustrates the sensitization of this response during later separations, and the bottom panel shows the effect of administering the anti-inflammatory naproxen (14 mg/kg) for 3 days just prior to the first separation. Naproxen-injected animals evinced lower levels of passive behavior than did vehicle-injected and non-injected controls during all three separations. Statistically significant differences are indicated by asterisks. Adapted from [64] (top panel) and [65] (bottom panel).

Preliminary support for this notion is afforded by experiments in young guinea pigs. When separated on several occasions between about 20 and 30 days of age, the animals exhibited a sensitized response. The number of 1-min intervals in which the young displayed crouching, eye-closure, and piloerection increased during a second separation 24 h later, and remained elevated during a third separation 10 days after the first [64] (Fig. 1 top panel). Moreover, injection with the anti-inflammatory agent naproxen for 3 days prior to the initial separation significantly reduced passive responding during all three separation episodes [65] (Fig. 1, bottom panel). These data indicate that cytokine release induced by the stress of separation can have a lasting effect on guinea pig behavior. More importantly here, they provide proof of principle that early innate immune activation in response to stressors has the capacity to shape later adaptive behavioral outcomes.

## Social influences on stress responses and behavioral phenotypes in older animals

*Social buffering of cortisol responses.* The presence of particular conspecifics continues to moderate stress responses in guinea pigs throughout the life span. These effects depend on the relationship of the animals to one another, the age at which they are tested, and the social conditions under which they live. Between the time of weaning (~3-4 weeks) and 2 mo of age, there appears to be little difference in the ability of female social partners to serve as buffers of the stress response. That is, the presence of the mother can still moderate HPA responses of both males and females, but so too can unfamiliar and familiar adult females [66,67]. In fact, unfamiliar females appear to be more effective than familiar, but unrelated females, at least for young males [68]. This effect may be related to the males’ increasing social attraction to other females at about this age [69].

In adulthood, dominant males housed in large social groups (~10 males, 12 females, plus offspring) in the laboratory form several small harems—as do wild guinea pigs (*C. aperea*) in their natural habitat [14,70]—and direct the vast majority of their socio-sexual behavior toward the females of their harem [14]. If such a male is placed alone into a novel enclosure, he shows a robust elevation of plasma cortisol levels. This cortisol response is reduced if the male's favored female from his harem is placed with him in the enclosure. However, there is no reduction in the male's response if he is tested with an unfamiliar adult female or even a female from the same social group, but not his harem [15,68]. This effect also has been observed in fully adult males housed with two females in standard laboratory caging. The female to which the male recently directed the most socio-sexual behavior reduced his cortisol response during exposure to a novel cage, whereas his other female cage-mate or an unfamiliar female did not [71]. Importantly, because adult females were almost always pregnant in the above studies, the results cannot be accounted for by the relative sexual attractiveness of the females at the time of testing. Rather, it appears that the social bond a male established with a particular female was the key to her ability to reduce the male's stress response.

A comparable situation exists for adult females. When a female from a large social group was exposed to a novel enclosure, the presence of the male with which she had expressed the most positive social behavior—and therefore was the presumed bonding partner—significantly reduced the female's plasma cortisol response, whereas the presence of a different male from the same colony produced only a nonsignificant reduction [72]. Likewise, when two females were housed with a male in standard laboratory caging, the male as well as the female with which a female was housed moderated her cortisol response, whereas an unfamiliar male did not [73]. Notably, the social interactions that the two females engaged in with each other, as well as with the male, were overwhelmingly positive, as is typical of females in the laboratory. In all, these studies demonstrate clear and selective social buffering of cortisol stress responses among both male and female adult guinea pigs. These effects appear strongest for those animals with the closest social relationship. In this way the effects parallel those seen in preweaning pups, where the attachment figure potently reduces stress responses of the pups.

*Cortisol and the shaping of behavior*. For males, there is one additional stage of development, and one at which social influences on HPA activity might be the most interesting of all. When males housed in large social groups first reach sexual maturity (~75-90 days of age), they have little chance of successfully breeding. Older, larger, and more-dominant males monopolize breeding opportunities with available females. During this time, the younger males of the colony attempt little courtship of females and avoid serious aggressive encounters with the larger males [15,74]. It is not until males reach about 7 mo of age that they are large enough to begin effectively challenging other males for breeding opportunities.

**Figure 2:**
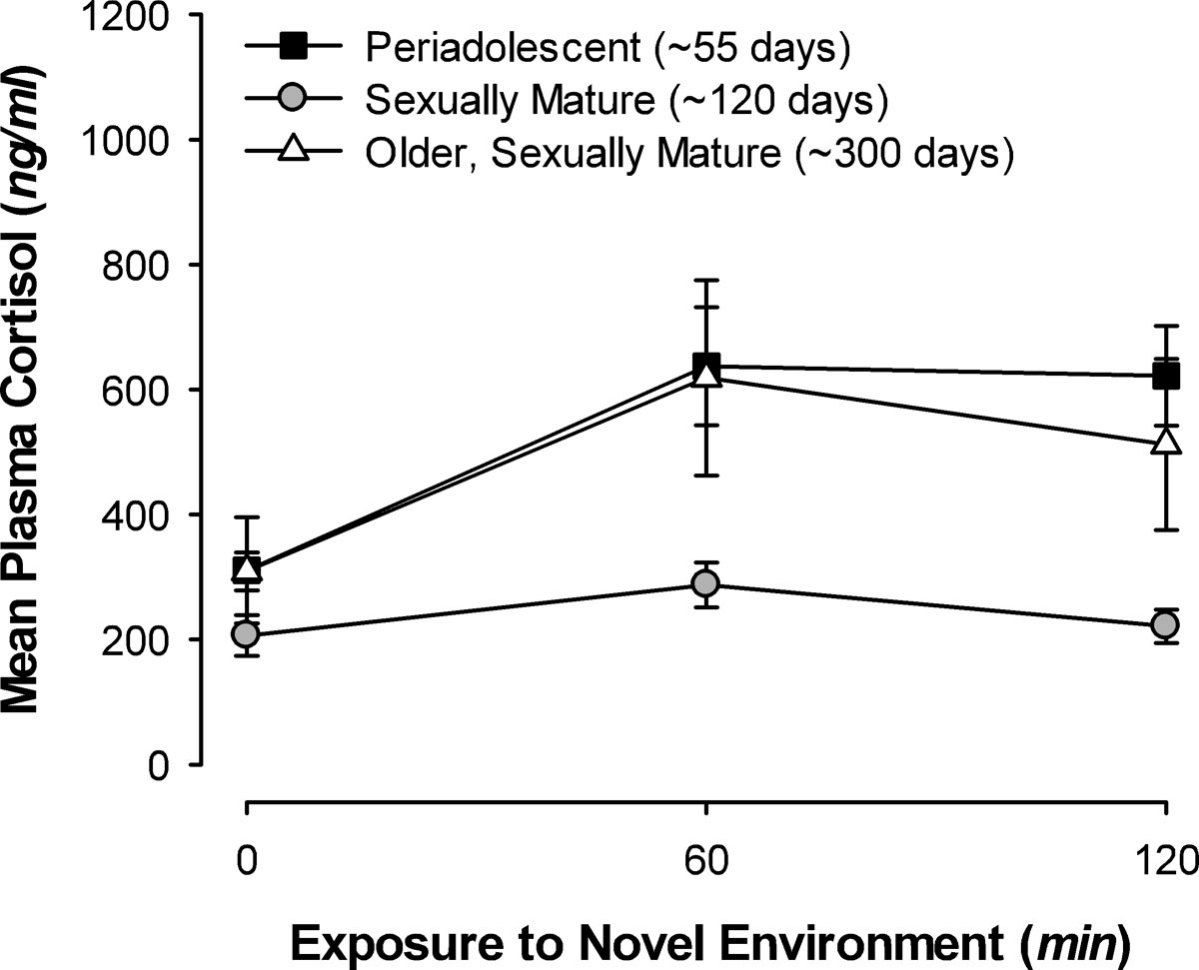
Mean (+/- se) plasma cortisol concentrations of male guinea pigs of different ages prior to, and 60 and 120 min following, isolation in a novel enclosure. The response of sexually mature males (~120 days) is suppressed relative to those of periadolescent (~55 days) and older, sexually mature males (~300 days). Adapted from [68].

Shortly after sexual maturity, males housed in large mixed age/sex colonies exhibit a dramatic change in HPA activity. When placed into a novel environment alone, they showed a nearly complete suppression of the plasma cortisol response [68]. Figure 2 compares the response to exposure to the test enclosure in males at this stage (sexually mature: ~120 days) with periadolescent (~55 days) and older, sexually mature (~300 days) males. In the absence of a typical cortisol response to the novel environment, there also was no social buffering effect during exposure. It is possible that this suppression of the cortisol response in males is related to emigration. While emigration has not yet been studied in *C. aperea*, anecdotal evidence suggests that males of about this age may disperse (Asher, personal communication). If so, a suppression in the physiological response to novelty might promote exploration of novel environments and thereby encourage emigration at this age.

Later studies showed that males housed with only one or two females rather than in large social groups showed no suppression of the cortisol response at this stage of development [71,75]. That is, the suppression of the cortisol response appeared to be socially mediated, but unlike the buffering effects described above, the effect did not depend on the presence of a single individual during exposure to the stressor. Instead, it was somehow related to the presence of multiple group members prior to stressor exposure.

A clue as to the mechanism of this effect was the observation that the colony-housed males of this age had higher circulating levels of testosterone than did similar-aged, pair-housed males [76]. Because testosterone has been shown to reduce cortisol levels in other species [77-79], this observation raised the possibility that social interactions with either males or females in the large colonies stimulated increased testosterone secretion, which in turn, suppressed the cortisol elevation. This hypothesis was supported by showing that provision of pair-housed males with limited additional stimulation (10 min/day for 20 sessions with an adult male or female) both raised circulating testosterone concentrations and reduced the cortisol response during exposure to novelty [80]. The ability of testosterone to suppress cortisol elevations in this situation was confirmed by showing that gonadectomy re-established the cortisol response of colony-housed males, whereas administration of testosterone reduced cortisol elevations of pair-housed males [81]. Thus, social conditions at this age can have complex and powerful influences on stress responsiveness.

The adaptive value of this social influence on stress responsiveness appears to be a function of its effect on behavior. Previous studies have found short-term increases in cortisol to stimulate aggression in other rodents [82,83]. In guinea pigs, introducing two adult males, each previously housed only with another female, leads to high levels of escalated aggression in which the loser must be removed to ensure survival [84]. In contrast, colony-reared males show little aggression when introduced to each other in this way [84]. Further, the colony-housed males easily integrate into another colony, whereas the elevated aggression of males lacking such social experience appears to prevent integration into a colony [85]. It seems then that the suppressed cortisol moderates the aggression of colony-reared males so that they can adopt a “queuing” strategy in which males refrain from direct confrontation with dominant males until they are large enough to effectively challenge them for access to breeding females. But what of the aggressive style of the pair-housed males—is it necessarily maladaptive? It has been proposed that the varying behavioral profiles of males housed under these different conditions may be accounted for by the “match/mismatch” hypothesis [86]. In the wild, the population density of *C. aperea* is known to vary, often dramatically [42,70]. In dense populations, a queuing strategy may lead to the greatest breeding success. Yet, in years in which the population is sparse, young males may have greater success with a more-aggressive strategy of defending and mating with any females they encounter. Even at times when population density is generally high, a male may locate a sparsely populated patch where the more-aggressive strategy would be effective. Thus, the social conditions under which young males live (dense population with a number of more-dominant males, or sparse population with few or no more-dominant males) may shape their neuroendocrine stress response, which then shapes the males’ behavior in ways that best match the particular environment in which they reside. Ongoing studies are providing direct tests of this prediction.

## Stability in the face of change

**Figure 3:**
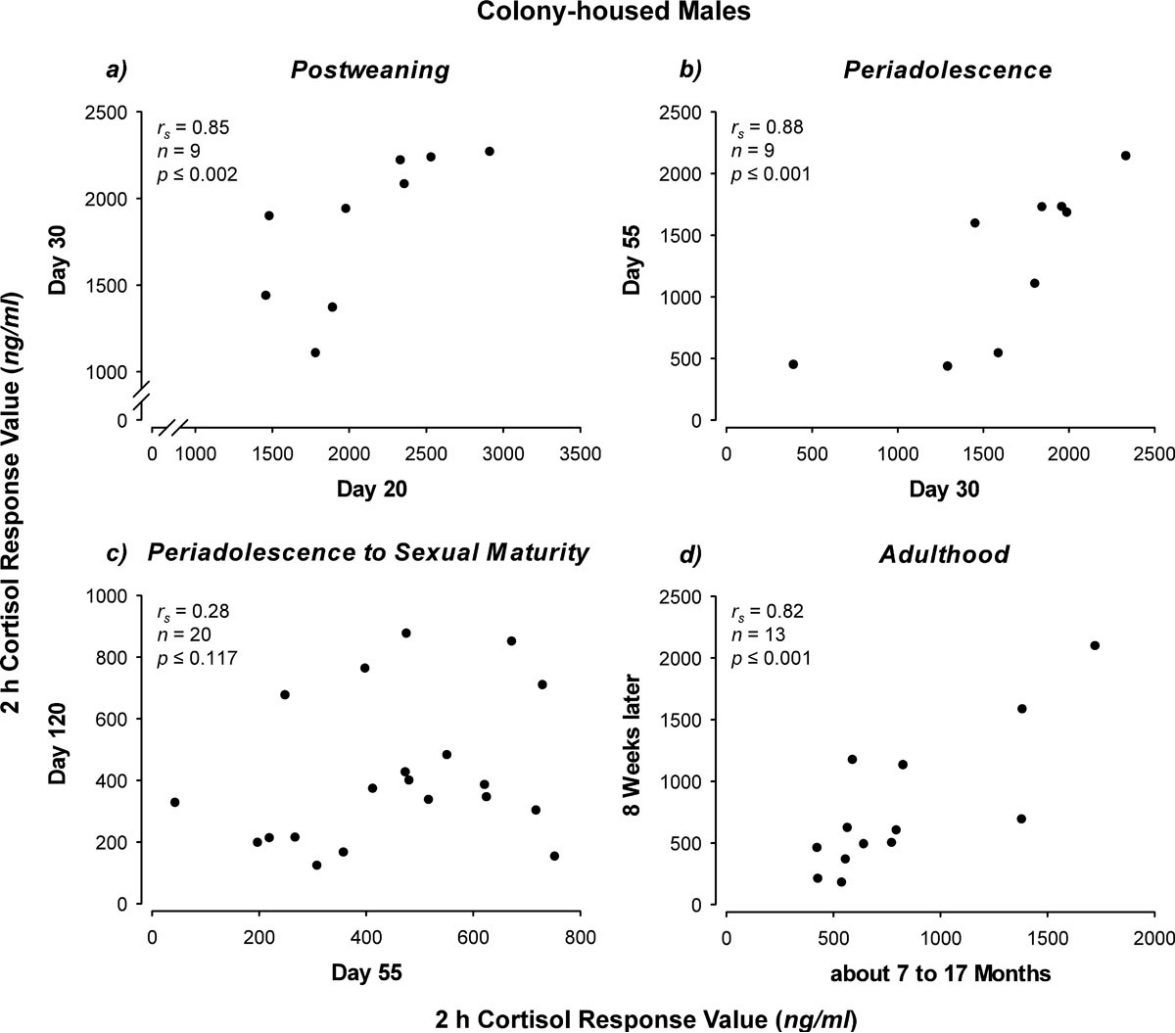
Cortisol responses during different life stages (a-d). Each dot represents plasma cortisol levels of a colony-housed male 2 h following placement into an unfamiliar enclosure at: a) 20 and 30 days, b) 30 and 55 days, c) 55 and 120 days, and d) about 7 to 17 months and 8 weeks later. Statistics: Spearman's rank correlation (one-tailed; parameters are indicated in the graphs). Redrawn and recalculated from [76,87-89], and unpublished data.

**Figure 4:**
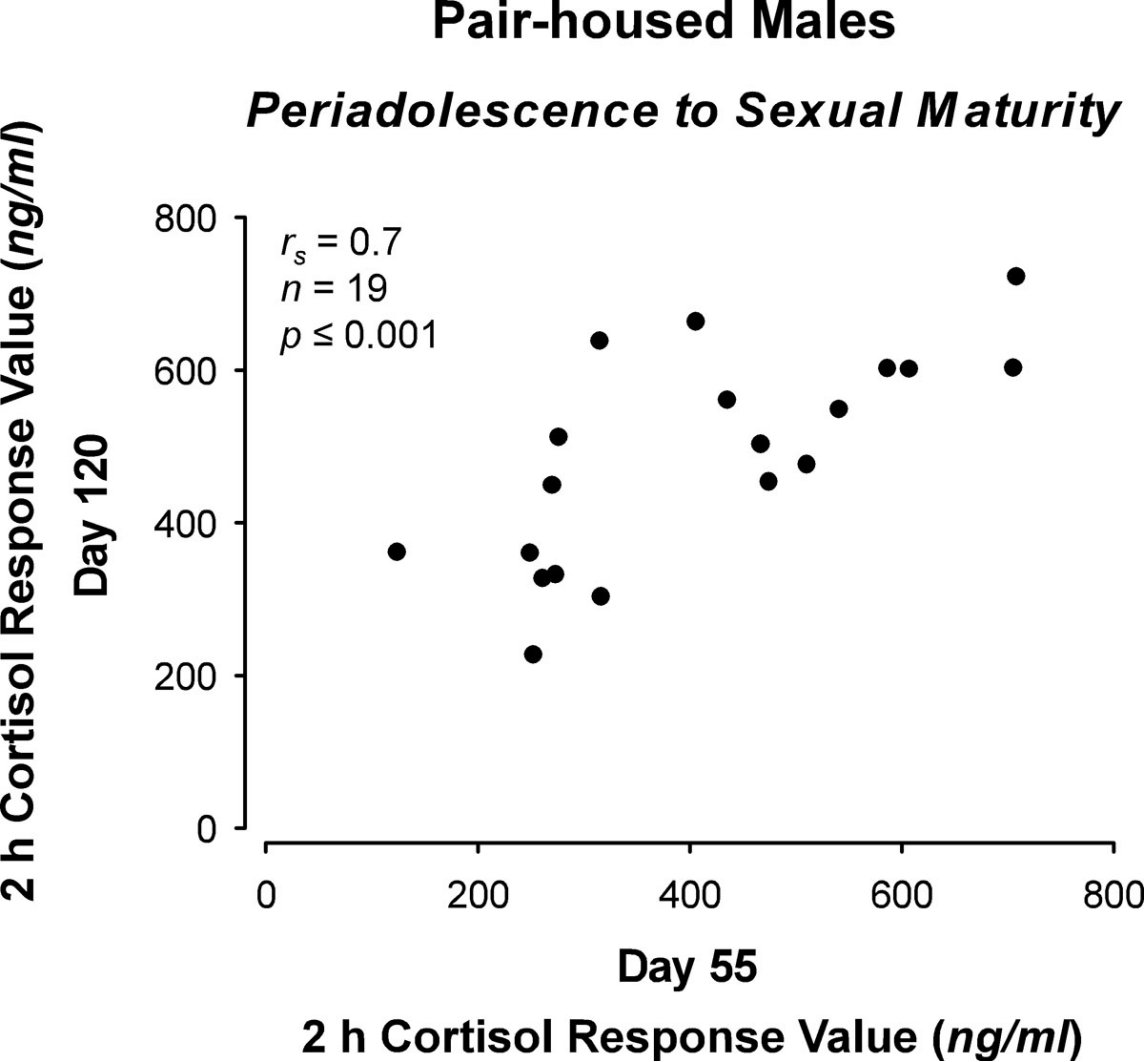
Cortisol responses of pair-housed males at about the time of sexual maturity. Every dot represents plasma cortisol levels of a male 2 h following placement into an unfamiliar enclosure at 55 and 120 days. Statistics: Spearman's rank correlation (one-tailed; parameters are indicated in the graph). Redrawn and recalculated from [76,80].

The preceding discussion suggests a highly plastic HPA stress response modulated by numerous social conditions at various life stages. However, this plasticity does not necessarily preclude a high degree of constancy as well. Most studies in this area focus on average outcomes in groups of animals treated one way or another. In contrast, we know very little about individual differences in stress responsiveness across development. In guinea pigs, a series of experiments provide insight into the stability of inter-individual differences in HPA reactivity during different life stages. When male colony-housed guinea pigs are repeatedly exposed to a novel enclosure, cortisol responses are remarkably stable during different life stages. That is, 2 h cortisol response values, for example, were found to be individually consistent from 20 to 30 days-of-age [87] (Fig. 3a) and from 30 to 55 days-of-age [88] (Fig. 3b), respectively. This stability was confirmed for older adult males (approximately 7 to 17 months) over an even longer retesting interval of about 2 months [89] (Fig. 3d). In contrast, no significant consistency in the cortisol response of colony-reared males was found from 55 to 120 days-of-age ([76] and unpublished data) (Fig. 3c)—the period in which males housed in this manner show socially mediated suppression of HPA reactivity, as described above. On the other hand, pair-housed males, which do not show suppressed HPA responsiveness, were observed to exhibit unambiguously stable individual differences in cortisol responses over exactly the same period ([76,80] and unpublished data) (Fig. 4). Taken together, it would appear that as the guinea pig progresses through the life span, the magnitude of the cortisol response is strongly affected by particular social stimuli at particular life stages; yet, during most stages individually stable differences in response magnitude persist. The potential adaptive value of such consistency in the individual cortisol stress response may be similar to that which has been proposed for behavioral traits in the “animal personality” (sensu, [90]) literature. When environmental conditions are unpredictable, as often is likely to be the case [91-93], one strategy might be to attempt to continually alter the nature of the behavioral profile in response to each environmental change. The costly investment required for this degree of flexibility may not pay off, however, if the environment continues to change more-rapidly than behavior can be adjusted. In such situations, it may be more beneficial to develop stable suites of behavioral responses that are effective in most situations, even though they may be ineffective in some [91-93]. This argument might be applied to the cortisol stress response since this essential trait of the biobehavioral profile (c.f., [89]) is critically involved in the control of behavior and provides the organism with energy to cope with challenge. A larger magnitude cortisol response would make more energy available, but at some cost; whereas a smaller cortisol response would make less energy available, but at a lower cost. It may also be a matter of how energy is made available; that is, by sympathetic or HPA activity. High sympathetic and low HPA activity is hypothesized to underlie a “proactive” coping style, and low sympathetic and high HPA activity is thought to support a “reactive” style [94].

## Conclusion

For those species for which environmental conditions can vary substantially from one generation to the next, there is great advantage in having early interactions with the inanimate or social environment alter the developmental trajectory of behavior so as to be adaptive for prevailing circumstances at later life stages. Early maternal influences on HPA responsiveness clearly represents a main mechanism through which such effects might be achieved. The guinea pig mother's influence on proinflammatory-mediated behavior offers a less-obvious possibility. Her absence and return readily evoke and suppress proinflammatory mediated behavior, respectively, and effects persist well after weaning. How these outcomes might affect fitness in differing environments remains to be determined, but the potential of the mother to adaptively shape later behavior of her offspring by unsuspected means should not be overlooked. Moreover, transformative social impacts are not limited to the earliest stages of life, as the consequences of colony- versus pair-housing on cortisol responses and behavior of maturing male guinea pigs vividly illustrate. The period around adolescence represents a chance to further “tune” influences originating with earlier experiences, or to institute completely new behavioral adjustments. Nevertheless, the modification of behavior to meet future environmental demands can only be an approximation. If a trait, such as the magnitude of HPA responsiveness, is adaptive for particular environments—or can shape behavior to be adaptive for those environments—it would appear to be useful for an individual to be also prepared for unpredictability and change of the future environments. Stable traits of the individual biobehavioral profile, such as those observed in the magnitude of the cortisol response of guinea pigs at different life stages, would seem to be an evolved mechanism to cope with such situations.

In this paper, we have focused almost entirely on laboratory findings. Indeed, many aspects of the natural history and ecology of wild guinea pigs remain unknown. For instance, we need to learn more about the ontogeny of social behavior in the natural environment, particularly perhaps in regards to dispersal. We also need to know more about how population density varies both temporally and spatially under different conditions. Once such information is in hand, it will be possible to more confidently make specific predictions about the adaptive value of particular findings, and to better place the laboratory results in the context of the natural environment.

## Declarations

We acknowledge financial support for this publication by the German Science Foundation (FOR 1232) and the Open Access Publication Fund of Bielefeld and Muenster University.

## Competing interests

The authors declare that they have no competing interests.

## Authors’ contributions

The initial draft was prepared by MBH. All other authors critiqued the initial draft and contributed intellectually to the final paper. TT prepared the figures. All authors approved the manuscript.
